# Prisoners' attitudes towards cigarette smoking and smoking cessation: a questionnaire study in Poland

**DOI:** 10.1186/1471-2458-6-181

**Published:** 2006-07-07

**Authors:** Alicja Sieminska, Ewa Jassem, Krzysztof Konopa

**Affiliations:** 1 Department of Pneumonology and Allergology, Medical University of Gdansk, Debinki 7, 80–952 Gdansk, Poland; 2 Department of Oncology and Radiotherapy, Medical University of Gdansk, Debinki 7, 80–952 Gdansk, Poland

## Abstract

**Background:**

In the last decade Poland has successfully carried out effective anti-tobacco campaigns and introduced tobacco control legislation. This comprehensive strategy has focused on the general population and has led to a considerable decrease in tobacco consumption. Prisoners constitute a relatively small part of the entire Polish population and smoking habits in this group have been given little attention. The aim of the study was to assess the prevalence of cigarette smoking in Polish male prisoners, factors determining smoking in this group, prisoners' attitudes towards smoking cessation, and to evaluate prisoners' perception of different anti-tobacco measures.

**Methods:**

An anonymous questionnaire including personal, demographic and smoking data was distributed among 944 male inmates. Of these, 907 men aged between 17 and 62 years (mean 32.3 years) met the inclusion criteria of the study. For the comparison of proportions, a chi-square test was used with continuity correction whenever appropriate.

**Results:**

In the entire group, 81% of the subjects were smokers, 12% – ex-smokers, and 7% – never smokers. Current smokers had significantly lower education level than non-smokers (p < 0.0001) and ever-smokers more frequently abused other psychoactive substances than never smokers (p < 0.0001). Stress was reported as an important factor in prompting smoking (77%). Forty-nine percent of daily smokers were aware of the adverse health consequences of smoking. The majority of smokers (75%) had attempted to quit smoking in the past. Forty percent of smoking prisoners considered an award for abstaining from cigarettes as the best means to limit the prevalence of smoking in prisons.

**Conclusion:**

The prevalence of cigarette smoking among Polish prisoners is high. However, a majority of smokers attempt to quit, and they should be encouraged and supported. Efforts to reduce cigarette smoking in prisons need to take into consideration the specific factors influencing smoking habits in prisons.

## Background

By the late 1980's only 10% of adult Polish men had never smoked placing Poland among those countries with the highest tobacco consumption in the world [1,2]. After introducing democratic changes and the market economy, Poland has become the target of the international tobacco industry being considered one of the best potential outlets. The tobacco industry quickly developed very aggressive tobacco advertising and promotions. Poland, however, successfully attempted to create a comprehensive tobacco control strategy, mainly through the introduction of new legislative measures [1]. The first anti-tobacco bill introduced in 1996, requested the biggest health warnings labels in Europe, and three years later all advertising in newspapers and on billboards were banned. This was accompanied by continuous increases in tobacco taxation with part of the taxes devoted to funding prevention efforts. Other anti-smoking measures included comprehensive information about health consequences of tobacco use, policies designed to prevent smoking in public places, workplaces, and other facilities, withdrawal of cigarette vending machines, forbidding the sale of loose cigarettes and sales to people under the age of 18.

All these measures have led to a gradual and steady decrease in tobacco consumption in both sexes and across age bands, except women aged 40–60 [3-5]. In the general population of adult men (mostly well-off and educated), the rate of 52% of daily smokers in 1990 decreased to 39% in 2002, and the rate of ex-smokers in the years 1990–1993 increased from 14% to 20% in the years 1997–2000. In the same period the prevalence of daily smoking in the general population of adult women dropped slightly from 26% to 24% [2,5]. However, these positive changes have not occurred in adults with a lower socio-economic status. The rate of daily smokers in this population is still higher compared to the general population: 53% among men and 30% among women [5].

Educationally and socio-economically disadvantaged groups are typically characterized by an increased criminality [6]. At present 80.000 people in Poland (2.7‰ of the general adult population) are incarcerated in the custodies or prisons [6,7]. The issue of tobacco smoking in this population has been neglected by public health sector, and health and economic benefits of smoking cessation in this community have not been estimated. Correctional facilities have not been taken into consideration in the national anti-tobacco strategies either. Very little attention has also been given to help prisoners stop smoking, although incarceration might be an opportunity to promote smoking cessation. On the other hand, prisoners' attitudes towards smoking cessation, their motivation to quit and awareness of a harmful smoking impact on health have not been investigated. Consequentially, it is not known whether prisoners are interested in participating in a smoking cessation or reduction program. Tobacco smoking in the correctional population has also been given little attention in other countries [8-13]. For example, in Australia, where tobacco control in the general community is of high priority, prison facilities have not been a target area for the state tobacco strategies [13]. In the USA, except for the implementation of some restrictions on smoking in some prisons, during the last 25 years no smoking cessation interventions in correctional systems have been reported in literature, whilst at the same time a number of anti-smoking interventions in other communities have been implemented [8].

The lack of data on tobacco smoking in Polish prisons prompted us to investigate this issue. We aimed to investigate the prevalence of smoking among male prisoners and their attitudes towards cigarette smoking and smoking cessation, as well as to establish their opinion on different anti-smoking measures. It could help to work out the most effective smoking cessation programmes addressed to this specific community.

## Methods

We used the data collected in the survey of Central Headquarters of Penitentiary Service (CHPS). Because the survey was conducted for internal programme development, ethical approval was not obtained prior to data collection. In the questionnaire form the respondents were informed about the aim of the study and possibility of "release of given information", as well as about the fact that participation in the questionnaire survey was totally voluntary and anonymous. A respondent's consent was taken into account while filling the questionnaire. Before analysing the data, the authors received permission from CHPS to use it in the present study.

The study sample was randomly selected among men incarcerated in prisons and jails of the Gdansk, Lublin and Lodz Penitentiary Districts in Poland. An anonymous voluntary questionnaire on cigarette smoking was distributed among 944 men. The questions referred to age, education, imprisonment status, smoking status, substance abuse, smoking initiation, the number of cigarettes smoked per day and changes in smoking habits in prison, factors enhancing smoking, the awareness of smoking consequences on health, the previous attempts to quit smoking, reasons to quit, and the causes of relapses [see Additional file 1].

The level of education was recorded as primary, vocational, secondary and university. According to an imprisonment status respondents were classified as provisionally detained, first sentenced and recidivist. To the first category of inmates, subjects held in pre-adjudication detention were classified (in the Polish legal system, the cumulative term of the pre-adjudication detention – until the detainee has been convicted – cannot exceed two years). Smokers (daily and occasional), ex-smokers and never-smokers were defined according to the WHO criteria: 1) a smoker was someone who, at the time of the survey, smoked any tobacco product either daily (daily smoker) or occasionally (occasional smoker), 2) ex-smoker was someone, who formerly had smoked daily, but, at the time of the survey, did not smoke at all, 3) never-smoker was someone, who either had never smoked at all or had never been daily smoker and had smoked less than 100 cigarettes (or the equivalent amount of tobacco) in his lifetime [14]. The assessment of substance abuse was carried out on the base of self-perception of substance use: "If you consider yourself as an abuser of any of the following substances, check one or more of the given patterns of your substance abusing: drinking excessive amount of spirits daily, drinking spirits once a week excessively, drinking too much wine or beer daily, tranquillisers, analgesics or other drugs abuse, narcotics, e.g., heroin, marijuana, cocaine, amphetamine abuse, incidental narcotic using and no substance abuse. According to the chosen pattern of alcohol drinking, taking into account the frequency, amount and type of alcohol beverages consuming, alcohol abusers were further recorded as: very excessive drinkers (daily spirits drinkers), excessive drinkers (drinking spirits excessively once a week) and mild drinkers (wine or beer daily drinkers).

The prisoners were also asked about their opinion on the given strategies for smoking cessation in prison. The question was: "How effective, in your opinion, each anti-smoking strategy would be: individual therapeutic meetings, group therapeutic meetings, system of awards for smokers abstaining from cigarettes (e.g. permission for additional visits or walks), anti-tobacco audio-visual measures – broadcasts or posters, nicotine replacement therapy, pharmacological agents (e.g., antidepressants)? Each strategy may be determined as not efficacious, low efficacious, efficacious or very efficacious".

### Statistical analysis

For the comparison of proportions, a chi-square test was used with continuity correction whenever appropriate. All reported values were two-sided. Statistical analysis was performed using the software package Statistica 6.0.

## Results

The response rate was 100%. However, 4% of questionnaires were not evaluated since they did not meet the criteria of the self-reported questionnaire. Finally, 907 men aged between 17 and 62 years (mean age 32.3 years) were included in the study. The distribution of the study population according to smoking status and selected characteristics is shown in Table 1.

A total of 841 respondents (93%) had a cigarette smoking history, including 736 (81%) current and 105 (12%) former smokers. The majority of ever-smokers (96%) reported smoking initiation before imprisonment. Only 4% of smokers started smoking in prison. Among smokers, 696 (95%) were daily and 40 (5%) occasional smokers. The distribution of daily cigarette smokers, according to selected characteristics of a smoking habit is shown in Table 2.

### Smokers awareness of the negative impact of smoking on health

Among prisoners with secondary and university education, 28% of smokers were aware of the negative impact of cigarette smoking on health, whereas among those with basic education – only 19%; (p = 0.033). The knowledge of negative effect of smoking on health was significantly more frequent in former smokers than in current daily smokers (63% and 49% respectively; p = 0.02).

### Factors influencing cigarette smoking and smoking cessation

The majority of smokers (75%) reported a stronger need to smoke while imprisoned than free, enhanced by a number of specific "prison" factors. The most frequent factors prompting cigarette smoking in prison are shown in Table 3. An equal proportion of smokers (75%) reported smoking cessation attempts ever in the past, both while being free (46%) and imprisoned (54%). The remaining 25% of smokers had never tried to stop smoking. The main reasons for quitting cigarettes are shown in Table 3.

The survey on awareness of smoking consequences on health demonstrated that quitters were more frequently aware of cigarette harmfulness than never-quitters, 55% vs. 33% respectively (p < 0.001).

Smokers who had never tried to quit cigarettes in the past were more likely to smoke over 20 cigarettes per day in comparison to those who attempted to stop smoking (26% and 17%, respectively; p = 0.0095). There were no significant differences according to the level of education (p = 0.139) and the imprisonment status between quitters and never-quitters (p = 0.19). Both groups did not differ in the prevalence of other substance abuse, except for very excessive and excessive spirit abusing. These patterns of alcohol abuse were more frequent among never-quitters than among quitters (36% and 28%, respectively; p = 0.04).

Over a half (52%) of quitters had attempted to stop smoking up to five times, and the remaining smokers had tried more than five times. The majority (64%) of former smokers (those who had successfully stopped smoking) achieved it in 1 to 3 attempts. Thirty-one per cent of subjects stopped smoking in the first attempt. Only nineteen smokers successfully stopped smoking during their stay in prison, composing 18% of former smokers. That number represented 2% of the surveyed ever-smokers.

The most frequent cause (67%) of failure in cigarette quitting was stress, either as a single cause, or in combination with other causes. Other factors included: alcohol drinking (25%), boredom (10%), depressed mood (8%), joy (5%) and yielding to one's persuasion (4%).

### Prisoners opinion on anti-smoking strategies

Forty per cent of smokers and ex-smokers considered the system of awards for smokers abstaining from cigarettes (e.g. permission to have additional visits or walks) the best measure to limit the prevalence of smoking among prisoners. Nicotine replacement therapy, individual therapeutic meetings, pharmacological agents (e.g., antidepressants), group therapeutic meetings and anti-tobacco audio-visual measures – broadcasts or posters – were assessed as very efficacious by 24%, 21%, 21%, 19%, 5% and 4% of smokers respectively. The estimation of the effectiveness of antismoking measures by prisoners was not related to the level of their education or to alcohol or substance abuse except for narcotics abusers, who more frequently considered an award system as the most effective (p = 0.02).

## Discussion

The community of prisoners differs from other social groups in terms of psychosocial factors, the level of education, alcohol and substance abuse, attitudes towards health, and lifestyle. All these factors account for the generally higher prevalence of tobacco smoking among prisoners, in comparison with a general population [8-11]. In Poland tobacco smoking in the correctional population has not been investigated so far, making this the first study on the subject.

In a randomly selected study group of male prisoners, accounting for nearly 1.2% of all Polish imprisoned males [6], we found extremely high rate of smokers. The data on the prevalence of smoking among female inmates remain still unavailable. Australian and American studies reported high rates of smokers among female correctional populations [8-10]. Among Queensland and Mississipi female prisoners the rates of smokers were 83% and 74%, respectively [8,9]. In American survey among female arrestees, the prevalence of smoking was also high, with the rate of 91% and 42% of daily smokers in New York and Los Angeles samples, respectively [10]. In all cases the rates of smoking were higher than in a general population. Similarly, in another Australian study, regarding both female and male correctional population, the rate of 79% of smokers was reported (three times more than that of a general population) [11].

In this survey, smoking habits were significantly more frequent among convicts (first sentenced and recidivists) than among provisional detainees. Moreover, smokers were more likely to be less educated, and this correlation was also reflected in other studies on general community populations [15,16] and Australian survey on female prisoners [9].

Almost all smokers in our sample started to smoke before imprisonment, and, in the light of three of Fagerström's criteria, the majority of them seemed to be strongly addicted to nicotine at the time of the survey [17]. The rates of smokers smoking 11–20 cigarettes daily and smokers having their first cigarette within 30 minutes after awakening were 59% and 79%, respectively.

It is considered, that people inclined to criminal and antisocial behaviours are likely to be predisposed to other forms of risky behaviours including tobacco and other substances abuse, mainly alcohol [18]. Moreover, alcohol drinking proved to be the strongest factor prompting relapses following smoking cessation [19], and ex-smokers seemed less likely to be alcohol abusers in comparison with current smokers [20]. Our study showed that among prisoners who defined themselves as alcohol and/or illicit drug abusers, almost 90% were current smokers. Alcohol was also an important factor prompting relapses in quitting. Smokers who had successfully quit smoking and never smokers significantly less frequently reported alcohol and/or other substances abuse than current smokers.

The analysis of factors that provoke smoking in our sample demonstrated the importance of the stress factor. On the other hand, in spite of specific stress resulting from imprisonment, the majority of smokers attempted to stop smoking under prison circumstances, but only 2% stopped smoking successfully while imprisoned. Surprisingly, the most important trigger to quit smoking was anxiety about health; reported by 46% of "ever-quitters". Although the awareness of harmfulness to health caused by smoking is generally not satisfactory in Polish correctional population, the commonness of this motivation to quit smoking habits could be in part explained with the Polish anti-tobacco legislation, which specifies that one-third of a pack of cigarette must be covered with warning labels.

The rate of never-quitters in our sample did not differ from that in other correctional populations, and represented nearly one-fourth of smokers [8,21]. However, the prevailing proportion of smokers had previously attempted to stop smoking at least one time, but unfortunately, most attempts to quit were unsuccessful. The rate of 12% of former smokers, namely subjects who quitted successfully, was relatively low in our sample. It is noteworthy, that every third successful quitter stopped smoking in the first attempt. The high rate of relapses during smoking cessation, mainly within 6 months, is common both in correctional and general populations [8,22]. It was previously demonstrated, that usually, people who cannot successfully quit smoking are particularly addicted to nicotine, and one of the most important factors determining relapses is alcohol use [19,22]. For that reason the community of prisoners, a substantial part of which consists of smokers usually abusing alcohol and strongly addicted to nicotine, is typically unsuccessful in quitting.

Quite a high proportion of quitters reflect a high willingness to stop smoking and indicate a great need for smoking cessation programmes addressed to them. On the other hand, the relatively high rate of never quitters in our study indicate that some policies should be adopted to improve smokers' information on tobacco consequences for health, and some counselling intervention added to prisons. The American survey conducted in Mississipi female prisons showed the high motivation to quit among current smokers, and the rate of quitters was 60% [8]. Almost two-thirds of smokers were interested in participating in a smoking cessation programme offered by the prison. In Poland, there are no cessation programmes addressed to prisoners and support offered to them is limited to an incidental advice by a physician examining newly admitted inmates. Thus, it should be of particular importance to add a minimal anti-smoking intervention to medical services in prison. A brief physician advice resulting in increasing quit rates up to 10% [23] seems an attractive strategy. To be effective, however, the medical staff should regularly attend smoking cessation workshops, which are, unfortunately, not organized in Poland because of the lack of funds.

Psychological studies indicate that smokers who successfully quit smoking were more frequently controlled by their intrinsic rather than extrinsic motives (e.g., the will to receive a reward) [24,25]. Smokers prompted by their intrinsic will to quit smoking were twice as frequently successful as smokers who were promised to receive a reward [25]. However, smoking prisoners in our study considered an award system the most efficacious method of cigarette quitting under prison circumstances, where they are deprived of the majority of usual pleasures accessible to free people. Probably, an award such as additional walking, physical training or meetings, including so-called intimate meetings with sexual partners, could be a strong motivational factor to quit or maintain the abstinence in the prison circumstances. This simple intervention encouraging smokers to stop smoking seems possible to be implemented in Polish prisons because of its low costs and high acceptance of smoking prisoners.

As far as the time for having a cigarette is concerned, prisoners in our sample generally smoked more cigarettes in the afternoon and in the evening than during the earlier hours of the day, which is not specific for strongly addicted smokers [17]. It is probably due to the typical Polish prison schedule – most daily activities like work, walking, physical training and meals are finished by four p.m. – making smoking cigarettes one of only very few available activities in the late hours. Thus, it may indirectly confirm the role of boredom as a factor increasing smoking in the second half of a day. Indeed, in our study prisoners reported boredom as an important trigger for smoking. The lack of activities results from many problems affecting Polish penitentiary system. First of all, prisons are over-crowded and since the year 2000 the number of prisoners exceeds the number of available places in prisons by ten thousands [6,26]. Besides, only 30% of prison cells have sufficient light to read and in the majority of prisons there are no community centres, often not even chapels [6,26]. The conditions to practise sport or any physical exercises in Polish penitentiary units are unsatisfactory, with few sports or exercise facilities [6,27]. As a result, the conditions are conducive to maintaining cigarette smoking, and potential efforts to reduce the demand for tobacco among prisoners may be less effective. Therefore, some regulations on smoking policy ban have been adopted in Poland to reduce the supply of cigarettes, as well as protect non-smokers against tobacco smoke. First of all, inmates sent to a Polish prison should be classified according to the smoking status, which allows to place non-smokers in smoke-free accommodation. Indoor smoking in Polish prisons is permitted only in individual cells and during meetings with visitors, and outdoor smoking – in walking yards. Smoking is banned during work and in all areas where non-smokers could be involuntary exposed to tobacco smoke, for instance community centres, chapels, gymnasium or hospital wards. However, the implementation of this policy has not been consistent. Prisoners who do not smoke are quite frequently accommodated with smoking people without any opportunity for non-smoking housing assignments. Inmates have opportunities to get cigarettes from the commissary or receive cigarettes in the parcels from friends or family members. The restrictive tobacco policies have not been effectively enforced in another countries either, including high income countries [28]. Experiences of these countries allow us to assume that total prohibition in Polish prisons would be difficult to enforce, due to several factors, including substantial costs and inmates' opposition to ban smoking in their cells.

The spectrum of potential tobacco policy options for prisoners is quite wide, although there are some difficulties in introducing them into this community [13]. The effectiveness of different anti-smoking interventions in a prison context has not yet been estimated in Poland. In our study, some forms of unstructured smoking cessation programmes like for instance provision of self-help materials or availability of NRT for sale to inmates, were perceived by smokers as low efficacious. Substitution of tobacco given to inmates at reception with NRT could be considered potentially effective, but at present still impossible to enforce in Polish prisons, mainly for economic reasons. There are also no structured quit programmes in Polish prisons, which normally consist of behavioural and pharmacological components and therefore are more cost-consuming.

Our findings highlight the crucial need for cessation programmes targeted to prisoners. Efforts to reduce cigarette smoking in prisons need to tackle both group and individual factors, and anti-tobacco programmes should be addressed to all aspects of smoking habits, including behavioural, psychological and biological processes governing addiction to nicotine. These programmes should be also adjusted to Poland's economic opportunities.

## Conclusion

Our findings suggest several implications for policy relevance. First, they indicate that prevalence of cigarette smoking among prisoners is high, particularly among the lower educated and substance abusing subjects. Secondly, the awareness of health consequences of smoking is strikingly low among prisoners, which calls for educational programmes adjusted to this community. Thirdly, the majority of smokers, mainly the better educated, attempt to quit smoking for many reasons. In the opinion of prisoners, the best measure to limit smoking in prisons is a system of awarding smokers for abstaining from smoking cigarettes. Finally, efforts to reduce cigarette smoking in prisons need to take into consideration the specific factors influencing smoking habits in prisons.

## Competing interests

The author(s) declare that they have no competing interest.

## Authors' contributions

AS conceived the study, participated in its design and coordination and drafted the manuscript. EJ participated in the design, coordination, and supervision of the study, and helped to draft the manuscript. KK performed the statistical analysis. All authors read and approved the final manuscript.

## Pre-publication history

The pre-publication history for this paper can be accessed here:



## Supplementary Material

Additional File 1 Provides detailed questions included in the questionnaire Click here for file

## Figures and Tables

**Table 1:** Distribution of the study population according to smoking status and selected characteristics

Characteristics	Total		Smoking status					
			
			Smokers		Ex-smokers		Never-smokers	
	
	No.	(%)	No.	%	No.	%	No.	%
Total number of subjects	907	(100)	736	81	105	12	66	7
Age								
≤ 20	85	(9)	74	87	4	5	7	8
21–30	372	(41)	296	79	51	14	25	70
31–40	253	(28)	203	80	28	11	22	9
41–50	156	(17)	132	85	16	10	8	5
> 50	41	(5)	31	75	6	15	4	10
Imprisonment status*								
Provisionally detained	319	(35)	238	75	37	11	44	14
First sentenced	227	(25)	193	85	21	9	13	6
Recidivists	351	(39)	297	85	45	13	9	2
Missing data	10	(1)	8	80	2	20	0	0
Education **								
Elementary or vocational school	648	(71)	544	84	65	10	39	6
Secondary/university	226	(25)	164	72	38	17	24	11
Missing data	33	(4)	28	85	2	6	3	9
Alcohol and/or illicit drug	500^†^	(55)	434	87	46	9	20	4
abuse ***								
Very excessive drinkers	122	(13)	107	88	10	8	5	4
Excessive drinkers	130	(14)	112	86	13	10	5	4
Mild drinkers	151	(17)	135	89	9	6	7	5
Drug abusers	95	(10)	76	80	16	17	3	3
Drug incidentally users	94	(10)	80	85	8	9	6	6
No substance abuse reported	362	(40)	261	72	55	15	46	13
Missing data	45	(5)	41	91	4	9	0	0

* p = 0.0002 comparing the rates of smokers and non smokers (combined ex- and never smokers) among subjects provisionally detained vs. others (first sentenced and recidivists)

** p < 0.0001 comparing the rates of smokers and non-smokers among subjects with secondary/university education vs. others (combined subjects with primary/vocational education and missing data).

*** p < 0.0001 comparing the rates of ever-smokers (combined current and former smokers) and never smokers among alcohol and/or illicit drug abusers vs. no substance abusers.

^† ^The number of 500 subjects reporting alcohol and/or illicit drug abusing does not reflect a total of the listed subcategories of abusers, because they are not exclusive.

**Table 2:** Distribution of daily cigarette smokers, according to selected characteristics of the smoking habit

Characteristic	Current smokers		Former smokers	
	
	No.	%	No.	%
Total No. of daily cigarette smokers	696	100	27	100
Age of smoking initiation:				
≤ 10	55	8	0	0
11–13	95	14	1	4
14–17	329	47	6	22
18–20	153	22	5	18
21–25	42	6	2	7
> 25	16	2	5	18
Missing data	6	1	8	30
No. of cigarettes smoked daily:				
1–5	12	2	6	22
6–10	128	18	6	22
11–15	116	17	4	15
16–20	293	42	3	1
21–40	126	18	1	4
> 40	15	2	0	0
Missing data	6	1	7	26
No. of pack/years:				
≤ 10	324	46	18	67
11–20	179	26	1	4
21–30	90	13	1	4
31–40	47	7	0	0
41–50	21	3	0	0
51–70	18	3	0	0
> 70	6	1	0	0
Missing data	11	2	7	26
Smoking the first cigarette:				
Within 30 min. after awakening	553	79	1	4
Later	131	19	4	15
Missing data	12	2	22	81
Smoking a greater No. of cigarettes:				
In the morning	310	44	3	11
In the afternoon	374	54	2	7
Missing data	12	2	22	81
Changes in smoking habit in prison:				
Smoking less cig/d. than at liberty	202	29	-	-
Smoking more cig/d.	231	33	-	-
Smoking the same No. of cig/d.	240	35	-	-
Missing data	23	3	-	-
Awareness of smoking negative impact on health:				
Aware smokers*	345	49	6	22
Smokers not contemplating whether smoking is harmful	227	33	3	11
Smokers denying health impacts of smoking**	103	15	0	0
Missing data	21	3	18	67

*smokers convinced about smoking harmfulness for health; **smokers convinced that smoking did not affect health in any way

**Table 3:** Most frequent factors promoting cigarette smoking during incarceration and smoking cessation (both in prison and at liberty)

Factors	No. of subjects	% ^†^
Factors promoting cigarette smoking in prison		
Missing family and close friends	435	66
The lack of freedom	381	57
Boredom	295	44
Anxiety about affairs to deal with at liberty	232	35
The lack of sex	206	31
Anxiety about the case and sentence	156	23
Qualms about the crime committed	116	17
Bad relations with the penitentiary staff	113	17
Bad relations with other prisoners	86	13
The lack of alcohol	70	10
The lack of narcotics	37	6
Other factors	21	3
Missing data	72	11
		
Factors promoting smoking cessation		
Anxiety about health	231	46
The fight with own weakness	142	28
The will to save some money	120	24
Limited access to cigarettes	109	21
Somebody's instigation	37	7
The will to gain an authority	10	2
Other (e.g., the bet with somebody, tobacco related disease in the family, physical or body-building activities)	32	6
Missing data	240	32

^† ^The percentages were estimated after excluding 72 and 240 smokers who had not replied to the questions regarding factors promoting cigarette smoking in prison and smoking cessation (both in prison and at liberty), respectively.

## References

[B1] Neroth P (2005). Stubbing out Communist habit. Lancet.

[B2] Zatonski WA, Boyle P, Gray N, Henningford J, Seffrin J, Zatonski W (2004). Tobacco smoking in Central European countries: Poland. Tobacco Science, Policy and Public Health.

[B3] Jaworski JN, Linke D, Przewozniak K, Zatonski W, Zatonski W, Przewozniak K (1999). Profilaktyka chorób odtytoniowych – narodowe kampanie zdrowotne. (Prophylaxis of tobacco related diseases – national health campaignes). Palenie tytoniu w Polsce: postawy, następstwa zrowotne i profilaktyka (Tobacco smoking in Poland: attitudes, consequences for health, and prophylaxis).

[B4] Zatonski WA, Willet W (2005). Changes in dietary fat and declining coronary heart disease in Poland: population based study. BMJ.

[B5] Zatonski W, Przewozniak K, Warszawa: PZH (2004). Cel operacyjny nr 3: Zmniejszenie częstości palenia tytoniu (Operative goal No. 3: A decrease in the prevalence of tobacco smoking). Kontrola spodziewanych efektów realizacji Narodowego Programu Zdrowia (Control of expected effects of the National Health Program fruition).

[B6] Headquarters of Penitentiary Service (2004). Podstawowe problemy więziennictwa (Fundamental problems of penology). Przegląd Więziennictwa Polskiego.

[B7] Central Statistical Office (2004). Rocznik statystyczny Polski (Statistical yearbook of the Republic of Poland).

[B8] Cropsey K, Eldridge GD, Ladner T (2004). Smoking among female prisoners: an ignored public health epidemic. Addict Behav.

[B9] Young M, Waters B, Falconer T, O'Rourke P (2005). Opportunities for health promotion in the Queensland women's prison system. Aust N Z J Public Health.

[B10] Durrah TL (2005). Correlates of daily smoking among female arrestees in New York City and Los Angeles, 1997. Am J Public Health.

[B11] D'Souza RM, Butler T, Petrovsky N (2005). Assessment of cardiovascular disease risk factors and diabetes mellitus in Australian prisons: is the prisoner population unhealthier than the rest of the Australian population?. Aust N Z J Public Health.

[B12] Centers for Disease Control (CDC) (1992). Cigarette smoking bans in county jails-Wisconsin, 1991. MMWR Morb Mortal Wkly Rep.

[B13] Awofeso N (2002). Reducing smoking prevalence in Australian prisons: a review of policy options. Appl Health Econ Health Policy.

[B14] World Health Organization (1998). Guidelines for controlling and monitoring the tobacco epidemic.

[B15] Droomers M, Schrijvers CT, Mackenbach IP (2002). Why do lower educated people continue smoking? Explanation from the longitudinal GLOBE study. Health Psychol.

[B16] Shohaimi S, Luben R, Wareham N, Day N, Bingham S, Welch A, Oakes S, Khaw KT (2003). Residential area deprivation predicts smoking habits independently of individual educational level and occupational social class. A cross sectional study in the Norfolk cohort of the European Investigation into Cancer (EPIC-Norfolk). J Epidemiol Community Health.

[B17] Fagerstrom KO (1978). Measuring degree of physical dependence to tobacco smoking with reference to individualization of treatment. Addict Behav.

[B18] Howard MO, Kivlahan D, Walker RD (1997). Cloninger's tridimensional theory of personality and psychopathology: applications to substance use disorders. J Stud Alcohol.

[B19] Shiffman SM (1982). Relapse following smoking cessation: A situational analysis. J Consult Clin Psychol.

[B20] Kohn CS, Tsoh IY, Weisner CM (2003). Changes in smoking status among substance abusers: baseline characteristic and abstinence from alcohol and drugs at 12-month follow-up. Drug Alcohol Depend.

[B21] Cropsey KL, Kristeller JL (2003). Motivational factors related to quitting smoking among prisoners during a smoking ban. Addict Behav.

[B22] Carmody TP (1993). Nicotine dependence: Psychological approaches to the prevention of smoking relapse. Psychol Addict Behav.

[B23] Cummings SR, Rubin SM, Oster G (1989). The cost-effectiveness of counselling smokers to quit. J A M A.

[B24] Cohen S, Lichtenstein E, Prochaska JO, Rossi JS, Gritz ER, Carr CR, Orleans CT, Schoenbach VJ, Biener L, Abrams D, DiClemente C, Curry S, Marlatt GA, Cummings KM, Emont SL, Giovino G, Ossip-Klein D (1989). Debunking myths about self-quitting: Evidence from 10 prospective studies of persons who attempt to quit smoking by themselves. Am Psychol.

[B25] Curry SJ, Wagner EH, Grothaus LC (1991). Evaluation of intrinsic and extrinsic motivation interventions with a self-help smoking cessation program. J Consult Clin Psychol.

[B26] Dobrzyniecki J (1998). Kultura fizyczna i resocjalizacja więźniów (Physical culture and resocialization of prisoners).

[B27] Rajzner A (1996). Kultura fizyczna w polskim systemie penitencjarnym (Physical culture in the Polish penitentiary system).

[B28] Puisis M (1998). Update on public health in correctional facilities. West J Med.

