# Supplementary material for: Prisoners' attitudes towards cigarette smoking and smoking cessation: a questionnaire study in Poland
Source: BMC Public Health. 2006 Jul 7;6:181. doi: 10.1186/1471-2458-6-181 (PMC1553442; doi:10.1186/1471-2458-6-181)
Supplement: Additional File 1 — Provides detailed questions included in the questionnaire [file 1471-2458-6-181-S1.doc]

Dear Respondent,

We kindly invite you to fill this questionnaire on tobacco smoking habits. Filling this questionnaire is voluntary. The questionnaire could help us to assess the prevalence of cigarette smoking among male inmates staying in prisons and custodies, as well as to determine the factors influencing smoking and smoking cessation during incarceration. This is an anonymous questionnaire, and you are asked to answer as honestly as you can. Filling it means that you give us your consent for using the given data in working out the anti-smoking programme.

1. I am ………………years old.

2. My education level is:

1. primary
2. vocational
3. secondary
4. university

3. I am:

1. provisionally detained in the custody
2. first sentenced
3. recidivist

4. If you consider yourself as an abuser of any of the following substances, mark one or more of the given patterns of your substance abusing:

1. Drinking excessive amount of spirits daily
2. Drinking spirits once a week excessively
3. Drinking too much wine or beer daily
4. Tranquillisers, analgesics or other drugs abuse
5. Narcotics, e.g., heroin, marijuana, cocaine, amphetamine abuse
6. Incidental narcotic using
7. No substance abuse

5. Do you smoke cigarettes at present, or have you ever smoked at least 100 cigarettes in your lifetime?

1. Yes
2. No

6. Please check one of the following, and give an answer for how long you have been smoking (in months or years):

1. I have smoked everyday for ………………………………………...years/months.
2. I have smoked not everyday for ……………………………………years/months.
3. I smoked in the past everyday for …………………………..years/months.
4. I smoked in the past not everyday for ………………………years/months.
5. I have never smoked tobacco.

**note! Only subjects who smoke cigarettes at present or who had smoked more than 100 cigarettes ever in the past are asked to fill the next part of the questionnaire**.

7. Please, give the age and the circumstances (before/during imprisonment) of your smoking initiation:

a) Before imprisonment at the age of…………….

b) During imprisonment at the age of…………….

8. Please give the number of cigarettes, which you smoke at present or smoked in the past daily…………………………………………

9. Do you smoke the first cigarette a day within 30 minutes after awakening?

1. Yes
2. No

10. Do you smoke a greater number of cigarettes…

1. …in the morning?
2. …in the afternoon?

11. Which of these sentences describe your beliefs about tobacco smoking?

a) Tobacco smoking is harmful for my health.

b) I have never contemplated whether smoking is harmful for health.

c) Tobacco smoking does not affect my health in any way.

12. Which of these sentences describe changes in your smoking habit in prison?

a) I smoke less cigarettes daily during incarceration than at liberty because of ..………………………………………………………. (*please give the reasons*)

b) I smoke more cigarettes daily during incarceration than at liberty.

c) I smoke the same number of cigarettes daily.

13. Do you consider a “ prison stress” a factor enhancing the need to smoke?

1. Yes
2. No

14. Which of the following factors enhance your need to smoke while imprisoned?

1. The lack of freedom.
2. Anxiety about the case and sentence
3. Qualms about the crime committed.
4. Bad relations with the penitentiary staff.
5. Bad relations with other prisoners.
6. Missing family and close friends.
7. Anxiety about your own affairs to deal with at liberty.
8. The lack of alcohol.
9. The lack of narcotics.
10. The lack of sex.
11. Boredom.
12. Other…………………………………………………………………………………….(*please give the examples*)

15. Have you ever attempted to quit smoking? (*Please, give the number of quit attempts*).

1. Yes, but only at liberty (……………….times)

If you chose this answer, reply please to the additional question:

*Do you consider smoking cessation in prison an additional stress resisting you to quit smoking during imprisonment?* A) Yes B) No

1. Yes, during incarceration and at liberty (…………………times)
2. No

16. Please, check the reasons for quitting attempts:

1. Limited access to cigarettes.
2. The will to save some money.
3. Anxiety about health.
4. The fight against your own weakness.
5. Somebody else’s instigation
6. The will to gain an authority
7. Other……………………………………………………………………………………

………………………………………………………………………………………………

17. If you have successfully quitted cigarette smoking, which of the following sentences describe this fact?

1. I have successfully stopped smoking at liberty.
2. I have successfully stopped smoking while imprisoned.

18. If you relapsed in quitting, please mark the reasons for it among the following:

1. Alcohol drinking
2. Stress
3. Yielding to one’s persuasion
4. Depressed mood
5. Joy
6. Boredom

19. Please check triggers for your tobacco smoking at liberty?

1. A pleasant taste and smell of smoke
2. Stress
3. The will to increase concentration
4. Relax
5. Having meals or drinking coffee
6. Drinking alcohol
7. Depressed mood
8. The will to resemble friends

20. How effective, in your opinion, each antismoking strategy implemented in prison would be? (*each strategy may be determined as not efficacious, low efficacious, efficacious or very efficacious*)

1. Individual therapeutic meetings………………………………………………
2. Group therapeutic meetings…………………………………………………………..
3. System of awards for smokers abstaining from cigarettes (e.g., permission for additional visits or walks)…………………………………………………………..
4. Nicotine replacement therapy…………………………………………………………
5. Pharmacological agents (e.g., antidepressants)…………………………………..

Thank you very much for filling the questionnaire.

Central Headquarters of Penitentiary Service, Warsaw
